# Views and perceptions about locally manufactured medicines in Ethiopia: a qualitative study of physicians, patients and regulatory authorities

**DOI:** 10.1186/s12913-018-3410-5

**Published:** 2018-08-08

**Authors:** Chalachew Alemayehu, Geoff Mitchell, Jane Nikles, Abraham Aseffa, Alexandra Clavarino

**Affiliations:** 1 Faculty of Medicine, The University of Queensland, P.O. Box: 78 Scott road, Brisbane, 4006 Australia; 2 UQCCR, The University of Queensland, Brisbane, 4029 Australia; 3 Armauer Hanson Research Institute, Addis Ababa, Ethiopia; 4 School of Pharmacy, The University of Queensland, Brisbane, Australia

**Keywords:** Local generic medicines, Lack of bioequivalence data, Perceptions, Ethiopia

## Abstract

**Background:**

Because of their cost, the use of locally produced, bioequivalent, generic drugs is universally recommended. In Ethiopia. while the government is committed to raising the market share and use of locally produced drugs, the process is hampered by the lack of a bioequivalence testing centre to strengthen the regulatory environment and deliver quality-assured local medicines. The purpose of this study is to assess the views and perceptions of key regulatory stakeholders, physicians and patients about locally produced generic medicines.

**Methods:**

A descriptive qualitative study, using focus group discussions and key informant interviews, was conducted. Five key informant interviews (two senior regulatory authority members and 3 institutional review board members) as well as 4 focus group discussions (2 with physicians and 2 with patients) were held. Data were analysed using an inductive, thematic process.

**Results:**

Four major themes emerged: awareness of lack of bioequivalence profiles associated with local medicines, perceptions about the quality and effectiveness of local medicines, quality and efficacy of imported medicines from developing countries and quality and efficacy of cheaper medicines. All institutional review board members were aware of bioequivalence issues. However, many physicians lacked detailed knowledge about bioequivalence, its clinical relevance and the lack of bioequivalence data for local medicines. All institutional review board members, physicians and male patients, but not female patients, were concerned about the quality and effectiveness of local medicines. Female patients were more confident about the locally produced drugs. In addition, some physicians and patients were not confident about the quality and effectiveness of cheaper drugs and drugs imported from developing countries. Government officials believed that local drugs are reliable.

**Conclusion:**

The success of promoting the use of inexpensive local medicines and changing the perception of the community depends not only on increasing the domestic market share held by local companies, but also on the capacity of the regulatory environment and companies to produce quality assured medicines and to overcome misconceptions. Among other initiatives, establishing an accredited bioequivalence centre in the country needs to be addressed urgently.

## Background

Around one third of the world’s population encounters difficulties in accessing medications because of high prices [1]. One of the strategies advocated to minimize the costs of medicines is the encouragement of greater use of generic medicines [2]. For example in the United States alone, $9 billion (11% of total prescription costs) was saved from 1997 to 2000 through the use of generic medicines [3]. Generic medicines are substitutes for original medicines. They are required to have the same quality and efficacy as the brand name medicines. Despite different regulatory guidance for marketing approval of generic medicines in different countries, bioequivalence testing is a fundamental regulatory requirement for approval of generic medicines [4]. Generic products are considered to be bioequivalent only if their rate and extent of absorption do not show a significant difference from the reference product [2].

The impact of the high cost of medicines is more pronounced in developing countries [1]. The United Nations recommends that the world’s poorest countries, such as Ethiopia, improve access to medicines through local production [5]. The current population of Ethiopia is around 101, 000, 000, which is equivalent to 1.35% of the total world population [6]. However, the local pharmaceutical industry in Ethiopia comprises only 15% of the total domestic market [7]. To address lack of access to affordable medicines by poor people, the government of Ethiopia is committed to raising the share of the domestic market held by local pharmaceuticals to 50% [7].

Despite the major advantages offered by generic medicines, their use all over the world is limited by lack of knowledge and negative beliefs among consumers and medical practitioners [8, 9]. Lack of knowledge on bioequivalence and regulation of generics were the main factors affecting healthcare stakeholders’, i.e., physicians, pharmacists and patients, attitudes towards generics substitution [10]. Therefore, many countries are implementing educational interventions, evidenced-based guidelines and generic drug policies that assist healthcare professionals to appropriately perform generics substitution [11–14].

Promoting generic drug substitution in developing countries like Ethiopia is a challenge for a functioning and reliable medicine regulatory system [15–18]. There are concerns about the quality of medicines in Ethiopia [19–21]. For instance, though *proof of bioequivalence* of a generic drug product is an essential *element* of *prequalification for effective generic drug substitution* [22], locally produced generic drugs in Ethiopia are approved without proof of bioequivalence [19]. This is due to lack of bioequivalence testing facilities in East Africa (including Ethiopia).

An insufficient medicine regulation system in Ethiopia could have a significant negative effect on the perceptions of physicians and patients about generic medicines. Moreover, use of generic drugs whose quality is not guaranteed, could lead to under-treatment and serious health consequences. For example, there is one documented case in which the Ethiopian medicine authority banned the production of one locally manufactured medicine after receiving claims about the ineffectiveness of the medicine from various health professionals [21]. Therefore, in addition to addressing supply issues (increasing the share of local pharmaceutical companies), identifying and managing demand (physician and patient) issues is important to promote the use of cost-effective locally-made drugs.

This study is part of a larger study assessing the feasibility of the implementation of pragmatic tests, namely N-of-1 tests, to confirm the therapeutic equivalence of marketed local drugs with imported drugs in Ethiopia. N-of-1 tests are double blinded, multiple cycle crossover trials, comparing a test treatment with a comparator. N-of-1 tests are indicated whenever there is substantial uncertainty regarding the comparative effectiveness of different treatments being considered for an individual patient.

The successful implementation of the N-of-1 equivalence test and the impact of the test on use of local medicines largely depends on the prior views held by the relevant stakeholders (patients, physicians, ethics and regulatory authorities) regarding the regulation and quality of local medicines. However, little is known about these perceptions.

What is known is that the few studies conducted on rational medicine use in Ethiopia report low level of knowledge and negative perceptions about generic medicines [23, 24]. Only 22.4% of patients were knowledgeable about generic medicine and barely 32% of patients had a positive attitude towards generic medicines.

A qualitative approach was chosen for this study because it allowed an in-depth exploration of the views and perceptions regarding the quality of local medicines and the unique contextual factors that shape the perceptions of people, specifically about the lack of proof of bioequivalence for marketed local medicines (which represent a cheaper treatment option in the usual care). Findings from this study will be used to inform the design, approval and implementation of a pilot study of N-of-1 therapeutic equivalence trials in Ethiopia.

Our research questions were:

- What are the perceptions of stakeholders about the effectiveness and quality of locally produced medicines? Do participants trust their local products? If not, why not?
- What are the views of stakeholders on bio-equivalence and lack of bioequivalence of local medicines compared with imported medicines?

## Method

This qualitative research examined perceptions about local medicines regulation and the quality of locally manufactured medicines. It was conducted using focus groups with medical practitioners and consumers as well as individual interviews with key informants responsible for the approval and conduct of N-of-1 tests in Ethiopia. The study was conducted in the All Africa Leprosy and Tuberculosis Rehabilitation and Treatment (ALERT) complex. It is one of the government owned institutes located in the capital city, Addis Ababa, in Ethiopia. The complex comprises the ALERT specialized hospital and the Armauer Hansen Research Institute (AHRI) and Training Centre. The study was approved by The University of Queensland ethics review committee (approval number: 2016-SOMILRE-0158) in Australia and by ALERT and AHRI ethics review committees locally (approval number: PO28/16).

### Focus groups

CA, with a trained facilitator, collected data using a digital-recorder and field notes. Both data collectors are male native speakers. Four focus group discussions, two with physicians and two with patients (one comprising male patients and one comprising female patients) were undertaken. The number of participants in each focus group ranged between six and eight, total 26. Interviews lasted from 24 to 45 min and focus groups lasted from 35 to 104 min.

A purposive sampling method was used to ensure inclusion of differing perspectives from the different stakeholders who would be involved during the pilot implementation of N-of-1 tests. Unifying the ALERT hospital and AHRI (a research facility), ALERT centre provides all-in-one flow of patients, clinicians and researchers. Because of our intention to pilot N-of-1 tests on hypertension, only hypertensive patients, who were being followed up at the ALERT hospital, were selected to participate in the patient focus group discussions.

The chronic care outpatient unit (CCOPU) in the hospital provides follow-up care for hypertensive patients. Most of these patients have at least a monthly visit schedule. We requested the CCOPU to obtain and invite a varied sample of patients during their hospital visit using the following inclusion criteria: adults between 18 and 70 years; both male and female; and have had at least 3 mos of follow up in the hospital. A total of 45 identified patients received invitation letters along with information sheets and informed consent forms. Finally, the CCOPU sent us the lists of 16 patients who signed the informed consent form. However, two of the patients chose not to participate because they were sick and two were too busy to attend the discussion. Thus, twelve patient participants, six females and six males were enrolled in the group discussions. Male and female group discussions were conducted separately to avoid conversations being dominated by men and encourage open dialogue among female participants.

To recruit physicians, we approached the hospital director to assist in recruitment of physicians who worked/are working in the chronic care unit. Physicians in the hospital often work in different departments - there is a constant rotation of work schedules across different departments. In collaboration with the hospital, the principal investigator physically contacted 16 physicians who have experience of working in chronic care unit. Then, invitation letters which included information sheets and informed consent forms were provided to them. Out of the 16 physicians invited, 14 of them responded to the invitation. Both patient and physician FGD schedules were arranged in consultation with participants and were conducted in AHRI.

### Key informant interviews

Five key informant interviews, with members of the institution review board (IRB) (*n* = 3) and Food, Medicine and Healthcare Adminsitration and Control Authority of Ethiopia (FHMACAE). (*n* = 2), were also conducted. The purpose of the key informant interviews was to obtain perspectives regarding the quality of local generics from both ethical and regulatory authorities, which were needed before designing the trial. IRB members were selected because of their role in the IRB of the ALERT complex. The two key informants representing the regulatory authority were senior officials involved in the approval of medicines and conducting of clinical trials in Ethiopia.

Data collection techniques among the key informant interviews were not consistent. While the key informants from FHMACA provided informed consent, they did not consent to the use of a digital recorder and detailed probing techniques. They also did not participate in the education sessions provided to other participants on the relevance and technical aspects of N-of-1 tests. We were only able to collect their very brief responses using field notes. All other participants engaged with no limitations.

The following issues were explored during the group discussions and interviews:

- What is a bioequivalence study? What are the regulatory and clinical uses of these studies?
- What are the issues associated with proof of bioequivalence of local medicines? Describe your understanding of reasons for lack of bioequivalence profiles for locally produced medicines in Ethiopia?
- Do you trust locally produced medicines? What is your perception about their quality and efficacy?

### Data collection and analysis

The interviews were conducted in the local language (Amharic), transcribed verbatim, and then translated into English. Brief notes were taken during the interviews with the respondents from the regulatory authority. On completion of each interview these notes were expanded. The expanded field notes were then incorporated with transcriptions, where available, to facilitate generation of themes. After familiarization with the data through repeated reading of the transcripts, a thematic framework [25] was developed using emerging ideas and a priori questions drawn from the objectives of the study. Specifically, transcripts were open coded for themes relating to interviewee views and perceptions, and included any other emerging themes, using an inductive, thematic process. The coding framework included four major themes.

### Trustworthiness

To enhance rigour, detailed description is provided of the sample, data collection, analysis and result. Maintaining reflective journal and documenting decisions were part of the research process to reduce the risk of being misled by our own experiences and expectations. To ensure different perspectives are represented, we examined similarities and differences across explanations and participant groups. Selected quotations from participants are included to allow the reader to judge interpretations and credibility of the analysis. Credibility was ensured by maintaining an audit trail. CM carried out the initial analysis. A sub-set of transcripts and field notes were analysed by AC, and themes were discussed with all research team members who had qualitative research expertise.

## Results

This study involved a total of 31 participants: 5 key informant interviews (two senior regulatory authority members and 3 IRB members) and 26 participants (14 physicians and 12 patients) in the focus group discussions (see Table 1). Participants’ ages ranged from 32 to 65 years. Of the total participants included [26], 11 were females. While two thirds of male patients had formal education, only one third of female patients had any formal education. Of the 3 ethics committee members, one had a senior physician role, one had a post-doctoral researcher position and the third one had an administration role at a government health office.

**Table 1:** Characteristics of study participants

Participants	Focus group	No. of people	Gender	Age	Education
Physicians	1	6	Male	35–59	Attend higher education [6]
	2	8	Male [6]	32–54	Attend higher education [8]
Patients	1	6	Male	51–6	Attend higher education [2] Secondary school[2] [1] Primary School [1]
	2	6	Female	48–57	Primary School Illiterate [4]
Ethics and regulatory authorities	Key Informant I interview	Overall [5] Ethics [3] Regulatory [2]	Female [3]	36–56	Attend higher education [5]

Using thematic analysis, we identified four major themes; awareness of lack of bioequivalence profiles associated with local medicines, perception about quality and effectiveness of local medicines, quality and efficacy of imported medicines from developing countries, and quality and efficacy of cheaper medicines. Quotes supporting each theme are presented, along with unique identification numbers of participants in brackets.

### Theme 1-awareness of lack of bioequivalence profiles associated with local medicines

Two key informants from the medicines regulatory authority, who are in charge of medicine approvals, were asked about the lack of bioequivalence requirements for locally produced generics. Neither of them was willing to comment on the lack of bioequivalence testing. One of them reported that they wouldn’t comment on national issues. They gave the impression that they believed the government follows its own direction to promote the availability of affordable medicines and that information on bioequivalence could affect the local companies and the public negatively.

Other IRB members and physicians were, however, willing to discuss their understanding of bioequivalence. They were asked whether they were aware of bioequivalence studies, the clinical relevance of such studies and the lack of proof of bioequivalence for locally manufactured medicines. All IRB members were aware of the bioequivalence requirement and its significance in determining therapeutic equivalence, as well as approval, of generic medicines. They were also aware that local medicines are marketed without any bioequivalence profile.

*‘Bioavailability of two medicines is studied using [bioequivalence] study. Before marketing generic medicine, companies conduct a [bioequivalence] study to test the standard of their medicine against the original product.’ (P3, IRB interview).*

*‘I consider [bioequivalence] study as one of the regulatory requirements especially to ensure quality… local pharmaceutical companies have gaps in this regard. However, there are ongoing efforts to make the test available in Ethiopia. (P1, IRB interview).*

The responses were varied among physicians. While the majority of the physicians were aware of bioequivalence studies, few had a detailed knowledge about its clinical relevance and whether there is a lack of bioequivalence regulatory requirements on local medicines or not. Few physicians reported awareness of the role bioequivalence studies play in relation to how generic medicines companies operate.

*‘Some years after the original medicine has (been) manufactured and used, the generic manufacturer could produce their (own) but they have to pass through bioequivalence study to compare with the brand medicine. After equivalence is confirmed, the company has the right to distribute the medicine in to the market. I also know that this is not the case in our country. ‘(P1, Physician FGD 1).*

*‘I know it is a medicine concentration test to prove the clinical efficacy of generic medicines’ (P4, Physician FGD 2).*

Some physicians reported a lack of bioequivalence as a reason for the prevailing concern that people had about local medicines.

*‘I mean the regulation and bioequivalence system in our country is very backward. We haven’t done much. To do bioequivalence[studies] is a better way to increase trust on local medicines.’ (P6, physician FGD 2).*

*‘I used to believe that our medicines have similar effects. But recently I recognized that local generic medicines are marketed before their equivalences is evaluated. ‘(P4, physician FGD 1).*

However, some physicians lacked detailed knowledge about bioequivalence studies and whether bioequivalence studies were a regulatory requirement or not.

*‘I know that there are different companies like Germany and Indian that produce similar medicines but I have no clear idea about the [bioequivalence] study’ (P2, Physician FGD 1).*

*‘Personally, I don’t have the information whether [bioequivalence] study is a regulatory requirement or not’* (P1, Physician FGD 2).

There were also some myths about the role of bioequivalence studies and their clinical relevance. For example, one physician pointed out that they are taught that, regardless of where a medicine is manufactured, it is the same, implying bioequivalence.

*‘We learnt that every medicine has similar bioavailability whether it is from Germany or India. Our senior staffs taught us that every medicines has equal effect. We are told that whether it is manufactured in Ethiopia, Western countries or Asia, Ceftriaxone is Ceftriaxone’.* (P2, Physician FGD 1).

One other physician noted that traditionally, and in practice, foreign manufactured medicines were preferred over locally manufactured medicines.

*‘I had never understood the science of bioequivalence but traditionally I know that we prefer Germany medicine or England’ (P2, Physician FGD 2).*

#### Reasons for lack of bioequivalence test on local medicines

All IRB participants and some physicians indicated that there was a lack of resources to establish a bioequivalence testing facility, as well as a lack of regulatory enforcement to ensure the bioequivalence requirements for local medicines are met.

*‘The country doesn’t have the resource to conduct bioequivalence study. The country is poor, I think that is the major reason’* (P2, IRB interview).

It is not only resources that are lacking; there is also a lack of regulatory enforcement to ensure the bioequivalence requirements for local medicines are met.

*‘In our setup, the regulation doesn’t enforce the implementation of bioequivalence testing. That is why local pharmaceutical companies are not forced to make this test’ (P7, Physician FGD 2).*

### Theme 2-perceptions about quality and effectiveness of local medicines

Participants across groups were asked about their perception of the quality and efficacy (or otherwise) of locally produced drugs.

The government officials interviewed believed that all medicines are approved for use after fulfilling a certain quality standards and they treat all medicines in the market, either local or imported, equally.

By contrast, most other participants across physician and patient groups reported that they were not confident about the quality and effectiveness of locally produced drugs, and all IRB members reported that they had concerns about the effectiveness of local drugs.

For example:

*‘Yes, there is a big concern regarding the quality of medicines. Because if you look at the medicines in the market you will notice a range of medicines from highly effective medicines to useless medicines with the same generic name being distributed for the clients. Some of the medicines are not more than a candy’* (P2, IRB interview).

*I think the Food, Medicine and Health Care Administration and Control Authority of Ethiopia (FMHACA) do some effort to regulate quality medicines from local companies but I don’t think it is adequate. Whether local medicines are effective or not is really an issue’* (P3, IRB interview).

Physician’s responses were similar to those of the IRB members:

*‘I am not confident with medicines produced in our country. I had used both generic and brand medicines to manage my patients. From my observation, some generic medicines are not effective and safe’* (P3, Physician FGD 1).

*‘We do have the concern on local medicines. It is something we repeatedly think of. Even personally, I think about it repeatedly… I don’t prescribe them to patients as long as they can afford to buy other medicines’.* (P4, Physician FGD 2).

The perception of patients about the effectiveness of locally manufactured medicines varied by sex. While most male patients reported perceiving a lack of quality and had a lack of trust in the local products, most female patients had relative confidence in local medicines.

These gender based differences can be seen in the following responses:

*‘Medicines which are produced in our country have quality problems. I do not know exactly but they are not curative as expected’* (P3, male FGD).

*‘We do not trust medicines that are manufactured in our country. When we take it, it is not as curative as the foreigners.’* (P2, male FGD).

On the other hand, female patients reported that they trust local medicines and were optimistic about taking local medicines. Views on medicines was related to level of education among female group, with a higher proportion with lower levels of education.

*‘What I am going to say is, ours (Ethiopian medicine) is better if it is right for all of us with the disease. Ours will be enough, all things from us’* (P2, female FGD).

If the medicine is the same with ours, ours will be good “yehagerun serdo behageru berie”^*^, if it is produced proportionally, we prefer ours’ (P3, female FGD). [^*^ *This phrase is a local proverb and it translates to “local products for the local people.”*]

#### Quality control

Some male patients commented on their expectations about the quality control of medicines and about getting the appropriate medicine for their need. They wanted physicians and the government to take control of the quality control issues.

*‘The physicians are responsible to recommend better medicines for us. Having a good medicine plays a key role in our health’ (*P3, male FGD).

*‘There should be an organization which makes a comparison study sustainably for the benefit of the community’* (P5, male FGD).

### Theme 3 – Variability in the quality and efficacy of imported medicines from developing countries

In addition to the quality of local medicines, several participants thought that developing countries in general were not capable of producing drugs equivalent to those produced in developed countries. Some physicians and patients believed that medicines which are imported from eastern countries, for example, India, were less effective than medicines imported from western countries. Some of the responses were surprising. One physician gave a practical example of the difference in effectiveness of drugs imported from India compared to those imported from Europe.

*‘There was a cryptoccocal meningitis patient who had frequent vomiting and we gave her plasil (metoclopramide) of Indian brand but the vomiting continued. However, with one injection of another plasil brand, the vomiting stops immediately because it was from Italy. After that I recognize the difference’* (P4, physician FGD 1).

*‘As of my informal knowledge and lesson from lectures, we are thought that Germany, England or other Western medicines had a better quality than medicines imported from India or China. I advise patients to use the western ones’* (P1, physician FGD 2).

*‘Honestly speaking, the product of Germany and British medicines are preferable. …they have a better capacity to produce better medicines.’* (P5, male FGD).

### Theme 4-quality and efficacy of cheaper medicines

The general belief that expensive medicines are superior contributed to the perception that the affordable local medicine options are not as effective as imported expensive medicines. . Though the medicines are in use in clinical practice, there were concerns about effectiveness.

*‘Cheaper medicines are dispensed in the pharmacy because patients cannot afford to buy other than these medicines. The price of medicines differs based on the country it is produced. Those medicines produced in our country are cheaper because they have lower efficacy than the others. This is what we think’* (P6, physician FGD 2).

*‘Whenever the medicine become cheaper and cheaper, the efficacy is also goes down. We have such thinking among clinicians’* (P6, physician FGD 2).

*‘Medicines which are imported from America, Germany and other rich countries are curative enough, people trust in these medicines. However, they are too costly. … Actually, there are certain medicines which are manufactured locally but they are not as curative (do not work as well) as medicines imported from those countries’* (P6, male FGD).

However, some participants did not agree that cheap drugs or drugs from eastern countries inevitably had lower quality than their western counterparts.

‘Drugs which are highly effective and useless drugs are in the market. Cheaper drugs could be as effective as the expensive ones. … When I work as a pediatrician in XXX Hospital, ceftriaxone is one of our drugs of choice for the treatment of infection. We usually prescribe drugs imported from Europe but most people couldn’t afford them so we would change it to Indian brand. Unbelievably, there is a big difference in price like 5 [Birr*] to 100[Birr] for the same drug. From clinical practice, I experimented and noticed that drugs from India have also the same effect. … So why would I prescribe expensive drug while the cheaper one has the same effect?’ (P3, IRB interview) [* The Birr is the local currency. There are approximately 16 Birr to $AU 1].

*‘Affordable medicines are preferable for us. Basically, there is a big difference in price. What we buy with 4 or 5 Birr may cost 150 to 160 Birr for foreign medicines’* (P1, female FGD).

## Discussion

Even in those countries with the resources and regulatory requirements in place to undertake bioequivalence studies, generic medicines are often viewed as inferior in quality and efficacy when compared to original brand medicines by medical practitioners [27–29]. One of the central debates is the issue of bioequivalence [27–29]. For this reason, several initiatives are underway to improve knowledge and perceptions of generic medicines and their regulation, and facilitate their prescribing in different countries [11–14].

This present study was conducted in an environment where generic medicines are commonly approved without proof of bioequivalence. All IRB members and some physicians were knowledgeable about bioequivalence and its clinical and regulatory significance for assuring the quality of generic medicines, as well as about lack of bioequivalence profiles of locally produced medicines. But many physicians lacked detailed knowledge about bioequivalence issues. Similarly, low levels of knowledge of the regulatory requirements for bioequivalence have been reported in previous studies conducted with medical practitioners from other developing countries [26, 30, 31], whereas 86% of physicians in United States of America (USA) reported that they could explain bioequivalence to their patients [32]. As mentioned above, there are initiatives in place to empower physicians and promote use of generic drugs in western countries [11–14].

This study also showed the presence of misconceptions by a few physicians about the role of bioequivalence studies and their clinical relevance, and demonstrated the limitations of medical education and the importance of clinical experience and clinical wisdom in Ethiopia.

Our study revealed that all IRB members and physicians have concerns about the effectiveness of local medicines. Physicians in this study did not believe that local generic medications approved by the government were therapeutically equivalent to their corresponding brand medication. This is in contrast to studies conducted in the USA and Saudi Arabia which both reported that 80% of physicians were confident about the effectiveness of local generic medicines [31, 32]. Although there were some misconceptions, the high level of negative perceptions about local medicines was primarily linked to the belief that there is insufficient medicine regulation in the country-and is not surprising given that medicine regulation in the country has been assessed as insufficient by WHO [19].

Contrary to the other groups, and although they would not comment about bioequivalence, regulatory authority officials believed that locally produced medicines have similar quality to that of comparable branded medicines. Their views may be shaped by their intention to foster the development and acceptance of local pharmaceutical companies, given the fundamental role of chepaer local drugs in the usual care. Local drugs have come to be used without appropriate bioequivalence test. Therefore, the regulatory authority need to first become aware of and acknowledge concerns of doctors and patients. Uncertainties regarding already approved drugs may not be high priorities to drug companies [33]. However, physicians and many patients were concerned regarding therapeutic equivalence of locally produced generic drugs. Acknowledging such differences between the priorities of society and industry is a critical step in addressing evidence gaps in local drugs.

For a number of the physicians, lack of trust in local medicines was part of the general belief that cheaper medicines have lower quality and efficacy than expensive ones. The most common reason for supporting local generics reported by both physicians and patients in this study, was cost. Other studies have also found that price differences influence physician prescribing of generics [34, 35].

Gender differences in beliefs about locally manufactured generic medicines were evident in this study. On the one hand, most male patients were concerned about quality and efficacy whereas most female patients were relatively confident about local medicines. This variation in perspective could be due to differences in the levels of education between males and females in Ethiopia. Until recently, females had much lower access to information and educations. This could likely impact females knowledge regarding regulatory requirements and risks associated with the use of drugs whose quality is not guaranteed. Moreover, female’s positive perception and optimism about taking local medicines may have been related to nationalism.

Some participants also had concerns about the efficacy of imported medicines from developing countries. Some physicians and patients believed that medicines imported from eastern countries, for example, India are less effective than medicines imported from western countries. Similar reservations about generic medicines manufactured in eastern countries, including India and China have been expressed by healthcare providers [36]. However, this perception is further reinforced by a recent study that found increasing numbers of poor quality medicines are being imported from India. According to the study, generic medicines sold in Africa by some Indian pharmaceutical medicine manufacturers are of substandard quality compared with the same medicines that the companies distribute for selling in India and non-African countries [37].

This study involved a range of stakeholders: medical practitioners, the regulatory authority and consumers. Although this study highlights a number of very important issues, there are also a number of limitations. The small number of key informant interviews, and focus group discussion participants who were purposely selected from a single hospital, could not represent the views of the whole population of stakeholders involved. The use of a male facilitator for the female patient focus group discussion may have influenced the willingness of the participants to express their views, particularly because females are not empowered in Ethiopian society. Female participants may have been more critical if a female facilitator had led the discussion. Because of a lack of detailed responses particularly from the regulatory authority, the findings reported here cannot be regarded as representing the views of the Ethiopian medicines regulatory authority. Moreover, the order in which questions were asked (questions on bioequivalence first followed by questions on quality of locally produced drugs) might have influenced responses - the more specific questions on bio-equivalence might have influenced responses to the more general questions on quality and trust of locally produced drugs. However, the insights gained across the various groups involved in the study provide a basis for generics manufacturers, policymakers and other stakeholders involved in establishing a bioequivalence centre in Ethiopia, to change perceptions and improve confidence in the use of local generic medicines. Strengths of the study included involvement of the broader stakeholders; drug prescribers, consumers and relevant decision-makers in drug regulation.

## Conclusions

In conclusion, the study showed that the majority of participants, including IRB participants, have little confidence in locally manufactured generic medicines. Unlike male patient participants, female FGD participants supported the use of local medicines. Brief responses from government officials indicated that local drugs are reliable; demonstrates the need for an open-discussion to deeply understand contextual motives of the views of the regulatory authority. To enhance the availability and use of effective, affordable local products, as well as increasing the local market share of these products, implementing measures that encourage physicians and patients to be confident about the use of local generic medication should be a priority for government. Raising physicians’ awareness about bioequivalence and other regulatory requirements that ensure quality has been a major initiative in many countries. Changing the perceptions of physicians and patients in Ethiopia requires an assurance that these cheaper local alternatives are interchangeable and can be used confidently. Ensuring availability of quality assurance mechanisms including bioequivalence tests, combined with information dissemination, are crucial to changing perceptions of both physicians and patients. The Ethiopian medicine registration guidelines document the need for proof of clinical equivalence, when pharmacokinetic bioequivalence data is not available [38]. One such practical measure is to adapt a pragmatic tool that can assess the therapeutic interchangeability of locally produced medicines. To this end, a study is currently being conducted to assess the therapeutic equivalence of locally and German-produced Enalapril using N-of-1 tests [39].

## Abbreviations

AAERC: ALERT Ethics Review Commitee
AHRI: Armauer Hansen Research Institute
ALERT: Africa Leprosy and Tuberculosis Rehabilitation and Treatment
FGD: Focus Group of Discussion
FHMACAE: Food, Medicine and Healthcare Administration and Control Authority of Ethiopia
IRB: Institution review board
USA: United States of America

## References

[1] World Health Organization (2008). Measuring medicine prices, availability, affordability and price components.

[2] King DR, Kanavos P (2002). Encouraging the use of generic medicines: implications for transition economies. Croat Med J.

[3] Haas JS, Phillips KA, Gerstenberger EP, Seger AC (2005). Potential savings from substituting generic drugs for brand-name drugs: medical expenditure panel survey, 1997–2000. Ann Intern Med.

[4] Davit B, Braddy AC, Conner DP, Lawrence XY (2013). International guidelines for bioequivalence of systemically available orally administered generic drug products: a survey of similarities and differences. AAPS J.

[5] Zarocostas J. World's poorest countries can improve access to medicines through local production, says United Nations. BMJ [Br. Med. J.]. 2011;342 10.1136/bmj.d3101 21593099

[6] United Nation (2015). World population prospect.

[7] Federal Democratic Republic of Ethiopia (2015). Investment Opportunity in the Pharmaceutical Sector in Ethiopia.

[8] Blatt CR, Trauthman SC, Schmidt EH, Marchesan S, LMd S, Martins JL (2012). General awareness and use of generic medication among citizens of Tubarão, state of Santa Catarina, Brazil. Cien Saude Colet.

[9] Wang D, Bakhai A (2006). Clinical trials: a practical guide to design, analysis, and reporting: Remedica.

[10] Dylst P, Vulto A, Simoens S (2013). Demand-side policies to encourage the use of generic medicines: an overview. Expert Rev Pharmacoecon Outcomes Res.

[11] Hassali MA, Alrasheedy AA, McLachlan A (2014). The experiences of implementing generic medicine policy in eight countries: a review and recommendations for a successful promotion of generic medicine use. Saudi Pharm J.

[12] Mossialos E, Mrazek M, Walley T (2004). Regulating pharmaceuticals in Europe: striving for efficiency, equity and quality: striving for efficiency, equity and quality: McGraw-hill education (UK).

[13] Kohl SH, Shrank WH (2007). Increasing generic drug use in Medicare part D: the role of government. J Am Geriatr Soc.

[14] Kanavos P, Taylor D (2007). Pharmacy discounts on generic medicines in France: is there room for further efficiency savings?. Curr Med Res Opin.

[15] Kaplan WA, Ritz LS, Vitello M, Wirtz VJ (2012). Policies to promote use of generic medicines in low and middle income countries: a review of published literature, 2000–2010. Health Pol.

[16] Mansfield PR, Mintzes B, Richards D. In: Toop L, editor. Direct to consumer advertising: British Medical Journal Publishing Group; 2004.

[17] Freudenberg N, Galea S (2008). The impact of corporate practices on health: implications for health policy. J Public Health Policy.

[18] Hassali MA, Wong ZY (2015). Challenges of developing generics substitution policies in low-and middle-income countries (LMICs). Generics and Biosimilars Initiative J.

[19] Assessment of medicines regulatory systems in Sub-Saharan African countries. An overview of findings from 26 assessment reports. 2010. Accessed 15 September 2015. Available from http://apps.who.int/medicinedocs/documents/s17577en/s17577en.pdf.

[20] Suleman S, Woliyi A, Woldemichael K (2016). Pharmaceutical regulatory framework in Ethiopia: a critical evaluation of its legal basis and implementation. Ethiopian journal of health sciences.

[21] Ethiopian Food Medicine and Health Adminstration and Control Authority (2010). Pharmacovigalance report.

[22] World Health Organization (2011). Marketing authorization of pharmaceutical products with special reference to multisource (generic) products: a manual for National Medicines Regulatory Authorities (NMRAs).

[23] Murray CJ, Frenk J (2000). A framework for assessing the performance of health systems. Bull World Health Organ.

[24] Berwick DM. Continuous improvement as an ideal in health care. Mass Medical Soc. 1989; 10.1056/NEJM198901053200110 2909878

[25] Ringe JD, Möller G (2009). Differences in persistence, safety and efficacy of generic and original branded once weekly bisphosphonates in patients with postmenopausal osteoporosis: 1-year results of a retrospective patient chart review analysis. Rheumatol Int.

[26] Alghasham AA (2009). Generic drug prescribing in Central Saudi Arabia: perceptions and attitudes of physicians. Ann Saudi Med.

[27] Fabiano V, Mameli C, Cattaneo D (2012). Perceptions and patterns of use of generic drugs among Italian family pediatricians: first round results of a web survey. Health Policy.

[28] Johnston A, Belitsky P, Frei U (2004). Potential clinical implications of substitution of generic cyclosporine formulations for cyclosporine microemulsion (Neoral) in transplant recipients. Eur J Clin Pharmacol.

[29] Toklu HZ, Dülger GA, Hıdıroğlu S (2012). Knowledge and attitudes of the pharmacists, prescribers and patients towards generic drug use in Istanbul–Turkey. Pharm Pract.

[30] Sharrad AK, Hassali MA, Shafie AA (2010). Generic medicines: perceptions of physicians in Basrah, Iraq. Australas Med J.

[31] Chua GN, Hassali MA, Shafie AA, Awaisu A (2010). A survey exploring knowledge and perceptions of general practitioners towards the use of generic medicines in the northern state of Malaysia. Health policy.

[32] Barrett L (2005). Physicians’ attitudes and practices regarding generic drugs.

[33] Giffin RB, Lebovitz Y, English RA. Transforming clinical research in the United States: challenges and opportunities: workshop summary: National Academies Press; 2010. 21210556

[34] Barrett JS, Batra V, Chow A (2000). PhRMA perspective on population and individual bioequivalence. J Clin Pharmacol.

[35] Zhao X-Y, Xu H-M, Zhou Q (2013). Sampling times and genotyping concerns in bioequivalence evaluation of branded and generic formulations. Ther Clin Risk Manag.

[36] Patel A, Gauld R, Norris P, Rades T (2012). Quality of generic medicines in South Africa: perceptions versus reality–a qualitative study. BMC Health Serv Res.

[37] Dyer O. Drugs exported from India to Africa are poorer quality than those sent elsewhere. BMJ: British Med J (Online). 2014;349 10.1136/bmj.g6017 25277463

[38] Food Medicine and Health Care Administration and Control Authority. Guideline for registration of medicines: Addis Ababa-FMHCAC; 2014.

[39] Alemayehu C, Mitchell G, Aseffa A, Clavarino A, McGree J, Nikles J (2017). A series of N-of-1 trials to assess the therapeutic interchangeability of two enalapril formulations in the treatment of hypertension in Addis Ababa, Ethiopia: study protocol for a randomized controlled trial. Trials.

